# Psychological state of a sample of patients with mood disorders during the first French COVID-19 lockdown

**DOI:** 10.1038/s41598-021-03037-w

**Published:** 2021-12-09

**Authors:** Emilie Olié, Jonathan Dubois, Myriam Benramdane, Sébastien Guillaume, Philippe Courtet

**Affiliations:** 1 Department of Emergency Psychiatry and Post-Acute Care, University of Montpellier, Montpellier, France; 2 IGF, CNRS, INSERM, Montpellier, France

**Keywords:** Risk factors, Depression

## Abstract

Since the beginning of the COVID-19 pandemic, evidence shows the negative psychological impact of lockdown measures in the general population. It is also important to identify predictors of psychological distress in vulnerable people, particularly patients with history of depressive episodes (the most prevalent psychiatric disorder), in order to adapt mental health strategies for future lockdown measures. This study aim was to (1) compare in 69 healthy controls (HC) and 346 patients with a major depressive episode in the two previous years (PP) self-reported psychological symptoms (depression, anxiety, insomnia, suicidal ideation, traumatic stress, anger) and living conditions during the first national French lockdown, and (2) identify predictors of significant psychological distress in PP. The levels of psychological symptoms were very low in HC compared with PP, independently of the living conditions. Half of PP had no psychiatric contact during the lockdown. Loneliness and boredom were independent predictors of depression, anxiety and insomnia, whereas daily physical activity was a protective factor. Virtual contacts protected against suicidal ideation. Our results highlight the need of specific strategies to target loneliness and boredom and to improve care access, including telepsychiatry. Longitudinal studies must investigate the COVID-19 pandemic psychological impact in clinical samples.

## Introduction

The global pandemic of the new coronavirus disease 2019 (COVID-19) that emerged in Wuhan, China, in December 2019, has spread rapidly worldwide^1,2^. To slow the infection rate, national lockdowns have been implemented almost everywhere. A timely review of the literature on previous epidemics alerted that quarantine measures might have negative psychological effects (post-traumatic stress, stress, anxiety, and depression symptoms)^3,4^. In agreement, many surveys published since the beginning of the pandemic highlighted the psychological effects of COVID-19-linked lockdown measures. In many European countries, depressive and anxiety symptom severity/frequency increased during the lockdown period^5–9^. These surveys in the general population showed that self-reported presence of a pre-existing mental health problem was an independent significant risk factor of negative psychological consequences of the lockdown^5,6,10^. The lockdown psychological impact in psychiatric patients also deserves more investigation. Some authors reported more severe depression, anxiety, and stress in individuals with self-reported affective disorders (i.e., bipolar disorder or major depressive disorder)^11^ and in individuals with mental illness compared with controls^12–14^. The lockdown psychological impact has been highest in people who reported a current mental disorder, followed by individuals with a past mental disorder, compared with the general population^7^. Patients with non-psychotic psychiatric illness were at high risk of experiencing higher levels of depression, anxiety, stress, insomnia, anger, irritability, and suicidal ideation compared with healthy controls^15^. Conversely, a study on individuals with severe mental illness (bipolar disorder and schizophrenia) did not detect any significant change in mood experiences, psychotic symptoms, and sleep duration^16^. Anxiety, depressive symptoms, and the practice of physical exercise were less frequent in patients with bipolar disorder and schizophrenia than in patients with depressive and anxious disorder^14^. Similarly, older adults with pre-existing major depressive disorder reported lower depression and anxiety during the pandemic^17^. In Spain, patients with psychiatric disorders coped well during the first few weeks of the pandemic, and more than 85% were able to enjoy their free time^12^. Altogether, these findings suggest the lockdown psychological impact may be different according the psychiatric history. Moreover, data are lacking on how psychiatric patients function during the COVID-19 pandemic. Therefore, we wanted to identify specific predictors of psychological distress in patients with past history of depressive episode (the most prevalent psychiatric disorder) to adapt mental health strategies in the perspective of future lockdown measures. Specifically, the aims of this study were: (1) to describe and compare psychological symptoms and conditions of lockdown in healthy controls and subjects with recent history of depressive episode, and (2) to identify predictors of significant psychological distress during the first national lockdown in France (from March, 17 to May 11, 2020). The whole population was confined at home, and only few professionals could go to their work place. Schools and universities were closed.

## Results

### Description of the sample (Table 1)

**Table 1:** Univariate comparison between HC and PP.

Variable	HC (N=69)	PP (N=346)	Statistic	DF	Effect size	p value
	Median [min;max] or N(%)	Median [min;max] or N(%)	Chi-2		Cliff’s delta/Odds Ratio 95% CI	FDR correction
Time lag between questionnaire and the beginning of lockdown (days)	31 [28;51]	38 [17;55]	14.28	1	0.29 [0.16;0.4]	< 0.001
**Sociodemographic** **variables**						
Men	16 (23.2)	83 (24)	0	1	1.04 [0.57;1.97]	0.99
Age (years)	37 [20;64]	39 [18;77]	0.141	1	0.03 [− 0.12;0.17]	0.79
High school graduation	53 (76.8)	178 (61.8)	4.851	1	0.49 [0.26;0.89]	0.05
Marital status						
Single	44 (63.8)	185 (53.8)	1.935	1	1	0.22
Couple	25 (36.2)	159 (46.2)			1.51 [0.89;2.61]	
Professional status						
Inactive	8 (16)	125 (51.2)	21.45	2	1	< 0.0001
Student	11 (22)	38 (15.6)			0.22 [0.08;0.6]	
Active	31 (62)	81 (33.2)			0.17 [0.07;0.37]	
**Psychopathology**						
Bipolar disorder	–	150 (44.5)				NA
Lifetime anxious disorder	–	207 (62)				NA
Lifetime eating disorder	–	51 (16.2)				NA
Lifetime Alcohol abuse/dependence	–	74 (22.2)				NA
Lifetime illicit substance abuse/dependence	–	51 (15.2)				NA
Lifetime history of suicide attempt	–	174 (50)				NA
**COVID related data**						
Suspected for COVID +*	7 (10.8)	13 (4.6)	NA	NA	0.4 [0.15;1.11]	0.12
Suspected Relatives for COVID+	17 (25.4)	71 (22.3)	0.144	1	0.84 [0.46;1.59]	0.79
Hospitalized or dead relatives due to COVID*	2 (3.9)	12 (4.3)	NA	NA	1.03 [0.26;7.36]	0.99
Fear of contamination for him/herself	4 [0;10]	3 [0;10]	3.707	1	− 0.15 [− 0.28; 0]	0.1
Fear of contamination for relatives	8 [0;10]	7 [0;10]	0.216	1	− 0.04 [− 0.18;0.11]	0.74
**Conditions** **of** **living** **during** **lockdown**						
Place						
At home	64 (92.8)	290 (84.1)	2.842	1	1	0.15
At other’s	5 (7.2)	55 (15.9)			2.36 [0.99;7.09]	
House facilities						
No outside	2 (2.9)	39 (11.3)	4.618	2	1	0.15
Balcony/patio	26 (37.7)	125 (36.1)			0.26 [0.04;0.95]	
Garden	41 (59.4)	182 (52.6)			0.24 [0.04;0.85]	
Surface						
< 50 m^2^	13 (18.8)	81 (23.4)	2.716	3		0.52
50–90 m^2^	23 (33.3)	131 (37.9)			0.92 [0.43;1.9]	
90–120 m^2^	16 (23.2)	75 (21.7)			0.75 [0.33;1.68]	
> 120 m^2^	17 (24.6)	59 (17.1)			0.56 [0.25;1.25]	
Presence of children (< 11 years old)	18 (26.1)	59 (13.1)	2.538	1	0.58 [0.32;1.09]	0.11
Activity organization						
Regular work	17 (24.6)	40 (11.6)	18.542	5	1	< 0.01
Short-time working	13 (18.8)	58 (16.8)			1.88 [0.82;4.4]	
Teleworking	9 (13)	47 (13.6)			2.19 [0.89;5.71]	
Student	12 (17.4)	41 (11.8)			1.44 [0.61;3.49]	
Unemployed	4 (5.8)	87 (25.1)			8.86 [3.02;33.29]	
Retired	14 (20.3)	73 (21.1)			2.2 [0.98;5.02]	
**Habitus** **during** **lockdown**						
Social support (Likert scale)	9 [0;10]	7 [0;10]	25.79	1	− 0.38 [− 0.49; − 0.26]	< 0.0001
Change in social support						
Decrease	4 (5.8)	54 (15.7)	5.23	2	1	0.12
As usual	53 (76.8)	225 (65.2)			0.33 [0.09;0.84]	
Increase	12 (17.4)	66 (19.1)			0.42 [0.11;1.3]	
Virtual contact						
Rarely	10 (14.5)	69 (19.9)	4.54	2	1	0.15
Several a week	23 (33.3)	144 (41.6)			0.91 [0.39;1.99]	
Every day	36 (52.2)	133 (38.4)			0.54 [0.24;1.13]	
Change in virtual contact						
Decrease	2 (2.9)	45 (13.1)	6.63	3	1	0.14
As usual	26 (37.7)	105 (30.5)			0.19 [0.03;0.69]	
Increase	27(39.1)	118 (34.3)			0.21 [0.03;0.74]	
High increase	14 (20.3)	76 (22.1)			0.26 [0.04;0.99]	
Mail contacts						
Rarely	10 (14.5)	66 (19.1)	4.67	2	1	0.15
Several a week	19 (27.5)	128 (37.1)			1.03 [0.43;2.31]	
Every day	40 (58)	151 (43.8)			0.58 [0.26;1.19]	
Change in mail contact						
Decrease	1 (1.4)	30 (8.7)	4.4	3	1	0.28
As usual	35 (50.7)	166 (48)			0.18 [0.01;0.88]	
Increase	21 (30.4)	98 (28.3)			0.18 [0.01;0.91]	
High increase	12 (17.4)	52 (15)			0.16 [0.01;0.91]	
Loneliness (Likert scale)	3 [0;9]	5 [0;10]	9.75	1	0.24 [0.09;0.38]	< 0.01
Change in loneliness						
Decrease	5 (7.2)	58 (16.8)	4.26	2	1	0.17
As usual	29 (42)	122 (35.4)			0.37 [0.12;0.94]	
Increase	35 (50.7)	165 (47.8)			0.42 [0.14;1.04]	
Going out from home						
Once a week or less	15 (22.1)	136 (39.4)	9.37	3	1	< 0.05
2 to 3 a week	19 (27.9)	94 (27.2)			0.55 [0.26;1.14]	
Every other day	10 (14.7)	39 (11.3)			0.43 [0.18;1.07]	
Every day	24 (35.3)	76 (22)			0.35 [0.17;0.71]	
Change in going out from home						
No change or increase	25 (36.8)	105 (30.5)	1.2	3		0.83
Low decrease	15 (22.1)	77 (22.4)			1.22 [0.6;2.52]	
Moderate decrease	17 (25)	94 (27.3)			1.31 [0.67;2.63]	
High decrease	11 (16.2)	68 (19.8)			1.46 [0.69;3.29]	
Physical activity						
Never	9 (13)	130 (37.6)	20.21	3	1	< 0.001
Once a week	12 (17.4)	55 (15.9)			0.32 [0.12;0.81]	
Several a week	21 (30.4)	93 (26.9)			0.31 [0.13;0.69]	
Every day	27 (39.1)	68 (19.7)			0.18 [0.07;0.39]	
Change in physical activity						
High decrease	11 (15.9)	74 (21.4)	1.86	4	1	0.83
Decrease	13 (18.8)	60 (17.4)			0.69 [0.28;1.66]	
As usual	26 (37.7)	123 (35.7)			0.71 [0.32;1.49]	
Increase	12 (17.4)	46 (13.3)			0.57 [0.23;1.42]	
High increase	7 (10.1)	42 (12.2)			0.89 [0.32;2.62]	
Boredom						
Never	30 (43.5)	75 (21.7)	15.96	2	1	< 0.001
Sometimes	25 (36.2)	142 (41)			2.26 [1.24;4.16]	
Often/continually	14 (20.3)	129 (37.3)			3.65 [1.84;7.54]	
Change in boredom						
Decrease or as usual	40 (58.8)	207 (60.2)	0.04	2	1	0.99
Increase	15 (22.1)	74 (21.5)			0.95 [0.5;1.87]	
High increase	13 (19.1)	63 (18.3)			0.93 [0.48;1.92]	
Alcohol use						
None	21 (30.4)	160 (46.6)	8.67	2	1	< 0.05
As usual	28 (40.6)	85 (24.8)			0.4 [0.21;0.75]	
Change of use	20 (29)	98 (28.6)			0.64 [0.33;1.26]	
Alcohol use for users						
Decrease	7 (14.6)	32 (17.5)	2.18	2	1	0.41
As usual	28 (58.3)	85 (46.4)			0.67 [0.25;1.64]	
Increase	13 (27.1)	66 (36.1)			1.12 [0.38;3.05]	
Tobacco use						
None	55 (80.9)	201 (58.6)	12.4	2	1	< 0.01
As usual	7 (10.3)	59 (17.2)			2.26 [1.03;5.74]	
Change of use	6 (8.8)	83 (24.2)			3.69 [1.64;10]	
Tobacco use for users*						
Decrease	3 (23.1)	22 (15.5)	NA	NA	1	0.5
As usual	7 (53.8)	59 (41.5)			1.18 [0.22;4.79]	
Increase	3 (23.1)	61 (43)			2.74 [0.44;17]	
Cannabis use*						
None	67 (98.5)	295 (89.1)	NA	NA	1	< 0.05
As usual	1 (1.5)	9 (2.7)			1.82 [0.33; 45.86]	
Change of use	0 (0)	27 (8.2)			NA	
Cannabis use for users*						
Decrease	0 (0)	13 (36.1)	NA	NA	1	0.34
As usual	1 (100)	9 (25)			NA	
Increase	0 (0)	14 (38.9)			NA	
Analgesic use						
None	57 (83.8)	236 (70)	8.34	2	1	< 0.05
As usual	11 (16.2)	70 (20.8)			1.52 [0.78;3.22]	
Change	0 (0)	31 (9.2)			NA	
Analgesic use for users*						
Decrease	0 (0)	11 (10.9)	NA	NA	1	0.18
As usual	11 (100)	70 (69.3)			NA	
Increase	0 (0)	20 (19.8)			NA	
**Psychiatric care**						
Psychiatric care						
None	69 (100)	184 (53.3)				NA
Tele-consultation	–	130 (37.7)				
Face-face	–	21 (6.1)				
Hospitalization	–	10 (2.9)				
Psychotropic drug use						
None	65 (95.6)	109 (31.6)	95.41	2	NA	<0.0001
As usual	2 (2.9)	161 (46.7)			44.37 [13.51;294.03]	
Change vs. usually	1 (1.5)	75 (21.7)			38.92 [8.4;918.14]	
Psychotropic drug use for users*						
Decrease	0 (0)	10 (4.2)	NA	NA	1	0.99
As usual	2 (66.7)	161 (68.2)			NA	
Increase	1 (33.3)	65 (27.5)			NA	
Pursuit of all medication						
No	6 (8.8)	25 (7.2)	84.85	2	1	<0.0001
Yes	16 (23.5)	265 (76.6)			4 [1.31;10.84]	
Not applicable	46 (67.6)	56 (16.2)			0.3 [0.1;0.76]	

Chi-square statistics, associated degree of freedom and Cliff’s non-parametric effect size or Odds Ratio and 95% confidence intervals are presented according to the test used (Kruskal–Wallis test, or Chi square test).

*DF* degree of freedom, *HC* healthy controls, *PP* psychiatric patients, *NA* not available, «*» Fisher’s exact test.

The sample for this observational study included 69 healthy controls (HC) and 346 psychiatric patients with a lifetime diagnosis of major depression episode (PP) who were contacted by e-mail approximately 1 month after the beginning of the first French lockdown. In a subsequent e-mail message, they received a form (sociodemographic data, living conditions, contacts, feelings of loneliness and boredom, physical activity, COVID-19 infection) and several psychometric tests (to assess depression, anxiety, anger, insomnia, traumatic stress and suicidal ideation) to be filled in (see “Materials and methods”).

They were mostly single [63.8% of HC (N = 44) and 53.8% of PP (N = 185)], women [76% of HC (N = 53) and 76.8% of PP (N = 253)], with a mean age of 38 years (no between-group difference for these variables). Compared with HC, PP had more often a lower level of education [23.2% of HC (N = 16) vs. 38.2% of PP (N = 110) without high school diploma, Chi2 = 4.851, df = 1, p = 0.05], and were more often inactive [16% HC (N = 8) vs. 51.2% PP (N = 125), Chi2 = 21.45, df = 2, p < 0.0001]. In the PP group, 44.5% (N = 150) had a diagnosis of bipolar disorder, 50% (N = 174) lifetime history of suicide attempt, 62% (N = 207) lifetime history of anxious disorder, 22.2% (N = 74) alcohol abuse or dependence, 15.2% (N = 51) illicit substance abuse or dependence, and 15.2% (N = 51) an eating disorder.

### Conditions of living and habits during the lockdown (Table 1)

Most participants were confined at home, with a surface > 50 m^2^, a garden or balcony, and few had children younger than 11 years of age. Moreover, 13% of participants (9 HC and 47 PP) were teleworking (33% of employed participants).

Feelings of boredom were less frequent in the HC than PP group [56.5% (N = 39) vs. 78.3% (N = 171), Chi2 = 15.959, df = 2, p < 0.001], without any reported change during the lockdown compared with the usual life in both groups. Compared with HC, PP reported higher loneliness level (Likert scale: 3, min–max: 0–9 vs. 5, min–max 0–10; Chi2 = 9.75, df = 1, p = 0.01) and lower social support (Likert scale: 9, min–max 0–10 vs. 7, min–max 0–10; Chi2 = 25.793, df = 1, p < 0.0001), but comparable frequency of virtual and written contacts (i.e. text messages, telephone and video calls, e-mails).

PP were less prone than HC to leave home [60.5% (N = 209) vs 77.9% (N = 53), Chi = 9.27, df = 3, p = 0.05] several times per week or to exercise [62.5% (N = 216) vs 87% (N = 60), Chi2 = 20.21, p < 0.01] at least once a week. In both groups, two third of subjects reported a decrease of the frequency of going out [63.3% HC (N = 43) vs 69.5% PP (N = 209), Chi2 = 1.2, df = 3, p = 0.83] and one third of participants reported a decrease in physical activity [34.7% HC (N = 24) vs 38.8% PP (N = 134), Chi2 = 1.86, df = 4, p = 0.83] due to the lockdown.

Few subjects suspected to be COVID-positive [10.8% HC (N = 7) vs. 4.6% PP (N = 13)], and about 25% had at least one close relative suspected of being COVID-positive [25.4% HC (N = 17) vs. 22.3% PP (N = 71), Chi 2 = 0.11, df = 1, p = 0.79]. Fear of COVID was not different between groups.

PP reported less frequently alcohol consumption compared with HC [53.4% (N = 183) vs. 69.6% (N = 48), Chi 2 = 8.67, df = 2, p < 0.05], but more frequently tobacco [41.4% (N = 142) vs. 19.1% (N = 13), Chi 2 = 12.40, df = 2 p < 0.01] or cannabis use [10.9% (N = 36) vs. 1.5% (N = 1), p = 0.04] and analgesic intake [30% (N = 101) vs. 6.2% (N = 11), Chi2 = 8.34, df = 2, p < 0.05]. One third of users reported an increase in their consumption of alcohol [27.1% (N = 13) HC vs. 36.1% PP (N = 66), Chi2 = 2.185, df = 2, p = 0.41] and tobacco [23% HC (N = 3) vs. 43% PP (N = 61), p = 0.5]. One third of PP (38.9%, N = 14) increased their cannabis use.

### Psychiatric care during lockdown (Table 1)

Since the beginning of the lockdown, 53.3% of PP (N = 184) had no psychiatric care, and 37.7% (N = 130) had telepsychiatry. In total, 31.6% of PP (N = 109) were not taking psychotropic drug, and 7.2% of PP (N = 25) interrupted usual medication. Among PP reporting psychotropic use, 4.2% (N = 10) reduced and 27.5% (N = 65) increased their consumption.

### Psychological outcomes during the lockdown (Table 2)

**Table 2:** Description of psychological outcomes.

Total score	HC (N = 69)	PP (N = 346)	Statistic	DF	Effect size	P value
	Median [min;max]	Median [min;max]	Chi-square		Cliff’s delta/odds ratio with 95% CI	FDR correction
PHQ-9	2 [0;9]	6.5 [0;27]	46.401	1	0.52 [0.41;0.61]	< 0.0001
GAD-7	2 [0;15]	6 [0;21]	28.664	1	0.41 [0.28;0.52]	< 0.0001
ISI	5 [0;18]	10 [0;26]	24.432	1	0.38 [0.24;0.5]	< 0.0001
IES-R	8 [0;26]	9 [0;40]	2.367	1	0.12 [− 0.02;0.26]	0.17
STAXI	13 [10;33]	15 [10;40]	9.583	1	0.23 [0.09;0.37]	< 0.01
Cut off	**N** **(%)**	**N** **(%)**				
**PHQ-9**						
< 10	69 (100)	223 (64.5)	33.177	1	1	< 0.0001
10_27	0 (0)	123 (35.5)			NA	
**PHQ-9** **item** **9**						
> **0**	1 (1.5)	92 (26.6)	19.171	1	21.19 [4.64;497.72]	< 0.0001
**GAD-7**						
< 10	67 (97.1)	250 (72.3)	18.336	1	1	< 0.0001
10_21	2 (2.9)	96 (27.7)			11.95 [3.65;79.26]	
**ISI**						
< 15	66 (95.7)	255 (74.1)	14.161	1	1	< 0.001
15_28	3 (4.3)	89 (25.9)			7.3 [2.62;31.46]	
**STAXI**						
< 13.5	39 (56.5)	145 (41.9)	4.404	1	1	0.07
≥ 13.5	30 (43.5)	201 (58.1)			1.8 [1.07;3.05]	
**IES-R**						
< 22	64 (94.1)	291 (84.6)	3.559	1	1	0.11
22_40	4 (5.9)	53 (15.4)			2.81 [1.1;9.75	
Psychological distress	11 (15.9)	189 (54.6)	32.945	1	6.25 [3.28;13.01]	< 0.0001

*DF* degree of freedom, *GAD-7* 7‐item Generalized Anxiety Disorder, *HC* healthy controls, *IES-R* Impact of Events Scale-Revised, *ISI* Insomnia Severity Inventory, *NA* not applicable, *PHQ-9* 9‐item Patient Health Questionnaire, *PP* psychiatric patients, *STAXI* *state* State-Trait Anger Expression Inventory.

High psychological distress was more frequent in PP than in HC [54.6% (N = 189) vs. 15.9% (N = 11), Chi2 = 32.94, df = 1, p < 0.0001]. All psychological outcomes were more severe in PP than HC, but for traumatic distress (Impact of Events Scale-Revised, IES-R) that was low in both groups (9, min–max: 0–40 in HC and 8, min–max: 0–26 in PP; Chi2 = 2.37, df = 1, p = 0.17). Current suicidal ideation (according to the suicidal item of Patient Health Questionnaire, PHQ-9) was reported by 26.6% of PP (N = 92).

For each psychological outcome (except anger), severe symptoms (current suicidal thoughts; 9‐item Patient Health Questionnaire, PHQ-9, score > 9; 7‐item Generalized Anxiety Disorder, GAD-7, score > 9; IES-R score > 22; Insomnia Severity Inventory, ISI, score > 14) were self-reported by fewer than five HC. Therefore, predictors of psychological outcomes were only analyzed in PP. Anger predictors were analyzed in the whole sample (HC and PP).

### Predictors of psychological outcomes (Table 3)

#### Anger (State-Trait Anger Expression Inventory, STAXI-state, score ≥ 13.5)

In the whole sample, anger was mainly predicted by frequent feelings of boredom (OR 95% CI 3.98 [1.78; 9.14]), and also by fear to be infected (OR [95% CI] 1.12 [1.01; 1.22]) and loneliness (OR [95% CI] 1.31 [1.19; 1.45]). Virtual contacts protected against anger (OR [95% CI] 0.34 [0.15; 0.72] for daily contacts, and OR [95% CI] 0.36 [0.167; 0.77] for weekly contacts).

**Table 3:** Predictors of psychological outcomes (multivariate models selected by Step AIC).

Characteristics	Levels	Anger (STAXI state ≥ 14)	Psychological distress	Depression (PHQ-9 > 9)	Anxiety (GAD-7 > 9)	Insomnia (ISI > 14)	Traumatic stress (IES-R > 21)	Suicidal ideation (PHQ-9 IS > 0)
		N = 343 AIC null = 473.5 AIC = 387	N = 249 AIC null = 346.28 AIC = 282.27	N = 246 AIC null = 315.16 AIC = 2 49.86	N = 246 AIC null = 332.51 AIC = 241.99	N = 251 AIC null = 287.01 AIC = 250.34	N = 251 AIC null = 218.83 AIC = 206.73	N = 251 AIC null = 287.01 AIC = 248.37
Group	PP vs HC	1.26 [0.65;2.47]	NA	NA	NA	NA	NA	NA
Time lag		1.01 [0.98;1.04]	1.02 [0.98;1.06]	1.00 [0.96;1.05]	1.01 [0.97;1.06]	1.03 [0.99;1.08]	1.02 [0.97;1.06]	0.99 [0.96;1.04]
Sex	Men vs women	1.11 [0.59;2.11]	0.85 [0.34;2.09]	0.85 [0.31;2.28]	1.40 [0.511;3.79]	0.94 [0.35;2.39]	**0.28** **[0.06;0.97]**	1.27 [0.47;3.28]
Age		1.01 [0.99;1.04]	0.99 [0.95;1.02]	0.99 [0.95;1.02]	0.98 [0.94;1.01]	**1.05** **[1.01;1.08**]	1.02 [0.98;1.05]	0.98 [0.95;1.01]
High school graduation		1.62 [0.93;2.87]	1.90 [0.86;4.33]	2.11 [0.93;4.95]	1.87[0.81;4.45]	1.36 [0.61;3.08]	1.29 [0.56;3.07]	**2.74** **[1.25;6.31]**
Bipolar disorder		NA	0.83 [0.39;1.76]	1.02[0.46;2.2]	1.47 [0.67;3.25]	0.71[0.31;1.58]	1.68 [0.74;3.86]	0.69 [0.31;1.48]
Lifetime anxious disorder		NA	**2.16** **[1.02;4.68]**	1.66 [0.74;3.82]	0.97 [0.44;2.12]	**3.67[1.61;9.07]**	1.26 [0.53;3.08]	1.34 [0.61;2.99]
Lifetime eating disorder		NA	1.88 [0.67;5.46]	1.29 [0.45;3.69]	1.57 [0.56;4.40]	1.73[0.65;4.54]	2.66 [0.95;7.44]	1.13 [0.43;2.94]
Lifetime alcohol abuse or dependence		NA	0.79 [0.28;2.16]	1.17 [0.44;3.12]	0.77 [0.28;1.99]	2.19 [0.89;5.41]	1.34 [0.46;3.61]	1.20 [0.45;3.12]
Lifetime illicit substance abuse or dependence		NA	2.32 [0.78;7.05]	1.08[0.35;3.32]	0.82 [0.28;2.28]	1.72 [0.61;4.75]	1.11 [0.34;3.32]	1.11 [0.38;3.13]
History of suicide attempt		NA	1.02 [0.49;2.1]	1.43 [0.67;3.11]	1.44 [0.64;3.29]	1.27 [0.59;2.77]	0.48 [0.21;1.08]	**2.79** **[1.31;6.22]**
Activity organization	Short-time working		0.36 [0.10;1.26]			1.32 [0.32;5.78]		
	Teleworking		0.83 [0.18;3.87]			1.28 [0.25;6.78]		
	Student		0.76 [0.19;3.03]			0.99 [0.20;5]		
	Unemployed		0.49 [0.14;1.65]			0.5 [0.13;2.04]		
	Retired		1.51 [0.43;5.33]			2.73 [0.76;10.97]		
House facilities	Balcony/patio							
	Garden							
House surface*	50–90 m^2^							
	90–120 m^2^							
	> 120 m^2^							
Presence of children (≤ 11 years old)								0.52[0.18;1.38]

Characteristics	Levels	Anger (STAXI state ≥ 14)	Psychological distress	Depression (PHQ-9 > 9)	Anxiety (GAD-7 > 9)	Insomnia (ISI > 14)	Traumatic stress (IES-R > 21)	Suicidal ideation (PHQ-9 IS > 0)
Fear of contamination		**1.12** **[1.01;1.23]**			**1.23** **[1.08;1.42]**		1.08[0.95;1.23]	
Fear of contamination for relatives			1.13 [0.99;1.3]	1.11[0.97;1.27]		1.06 [0.93;1.22]		
Going out from home*	2–3 a week		**2.46** **[1.02;6.16]**	2.28[0.87;6.13]				
	Every other day		0.31 [0.08;1.1]	0.57[0.11;2.35]				
Physical activity*	Every day		2.4 [0.90;6.59]	**5.36** **[1.87;16.41]**				
	Once a week		0.27 [0.09;0.75]	0.71 [0.25;1.98]	0.46 [0.16;1.3]	0.41 [0.13;1.17]		**0.26** **[0.08;0.79]**
	Several times a week		**0.39** **[0.16;0.93]**	**0.39** **[0.15;0.97]**	**0.34** **[0.13;0.82]**	0.6 [0.25;1.41]		0.69 [0.28;1.63]
Virtual contact*	Every day		**0.14** **[0.04;0.41]**	**0.2** **[0.05;0.64]**	**0.17** **[0.04;0.57]**	**0.28** **[0.08;0.88]**		0.66 [0.22;1.87]
	Several a week	**0.36** **[0.17;0.77]**						0.47 [0.18;1.22]
Social support	Every day	**0.34** **[0.15;0.72]**						**0.23** **[0.08;0.65]**
			0.91 [0.8;1.04]	0.9 [0.78;1.03]		0.94 [0.83;1.06]		0.94 [0.82;1.07]
Loneliness		**1.31** **[1.19;1.45]**	**1.20** **[1.05;1.38]**	**1.2** **[1.05;1.39]**	**1.28** **[1.12;1.49]**		**1.32** **[1.15;1.53]**	**1.33** **[1.17;1.53]**
Boredom *	Sometimes	1.15 [0.61;2.19]	0.94[0.37;2.37]	1.61 [0.55;5.01]	1.27 [0.42;4.13]	0.99 [0.35;2.96]		
	Often/continually	**3.98** **[1.78;9.14]**	2.6 [0.80;8.62]	**6.94** **[2.19;24.09]**	**3.71** **[1.15;12.94]**	**7.57** **[2.65;23.99]**		
Alcohol use	As usual				2.71 [0.99;7.62]			
	Change				3.39 [1.4;8.5]			
Psychotropic drug use	As usual		1.73 [0.82;3.72]	1.48 [0.62;3.59]	1.71 [0.72;4.26]			1.53[0.64;3.8]
	Change		**7.13** **[2.46;22.9]**	**5.86** **[2.15;17.03]**	**4.41** **[1.66** **;12.28]**			**4.7** **[1.79;13.09]**

Continuous covariables are age, loneliness (Likert scale), social support (Likert scale), fear to be contaminated (Likert scale), fear of contamination for relatives (Likert scale).

Socio-demographic variables are forced in the step AIC process as well as lifetime psychiatric diagnoses when considering only PP. N, AIC for the null models (including only the intercept) and AIC for best models are presented.

Anger state is evaluated for the whole sample (HC and PP) without taking into account psychiatric diagnoses. Other psychological outcomes were evaluated for PP only.

*STAXI* state-trait anger expression inventory, *PHQ* *9* Patient Health Questionnaire, *GAD-7* Generalized Anxiety Disorder, *IESR* impact of Events Scale–Revised, *ISI* Insomnia Severity Inventory, *PHQ9* *IS* item of PHQ-9 assessing suicidal ideation.

*Base levels (intercept) for qualitative variables with more than two levels are : regular work, house without facilities, surface < 50 m^2^, to go out from home once a week or less, no physical activity, poor frequency of virtual contact, no alcohol consumption, no psychotropic drug use and no boredom.

#### Depression (PHQ-9 score > 9)

Frequent feelings of boredom and change in psychotropic drugs were the main predictors of moderate-severe depression (OR [95% CI] 6.94 [2.19; 24.09] and 5.86 [2.15; 17.04] respectively) in PP. Patients going out every day were more likely to report depressive symptoms than those staying at home (OR [95% CI] 5.36 [1.87; 16.41]). Loneliness also was predictive of depression (OR [95% CI] 1.2 [1.05; 1.39]). Weekly sport practice was protective against depressive symptoms (OR [95% CI] 0.39 [0.15; 0.97] for several times/week, and OR [95% CI] 0.2 [0.05; 0.64] for every day).

#### Suicidal ideation (suicidal item of PHQ-9 > 0)

Suicidal ideation was predicted by psychotropic drug changes (OR [95% CI] 4.70 [1.79; 13.09]), history of suicide attempt (OR [95% CI] 2.79 [1.31; 6.22]), high education level (OR [95% CI] 2.74 [1.25; 6.31]), and loneliness (OR [95% CI] 1.33 [1.17; 1.53]. Daily virtual contacts were protective against suicidal ideation (OR [95% CI] 0.23 [0.08; 0.65]).

#### Anxiety (GAD-7 score > 9)

The most predictive factor of moderate anxiety was a self-reported change in alcohol consumption (OR [95% CI] 3.39 [1.40; 8.5]) and in psychotropic drug use (OR [95% CI] 4.41 [1.66; 12.28]), followed by frequent boredom (OR [95% CI] 3.71 [1.15; 12.94], loneliness (OR [95% CI] 1.28 [1.12; 1.49]), and fear to be infected (OR [95% CI] 1.23 [1.08; 1.41]). Practicing sport several times per week was associated with lower anxiety levels (OR [95% CI] 0.34 [0.13; 0.82] for several times/week, and OR [95% CI] 0.17 [0.04; 0.57] for every day).

#### Traumatic stress (IES-R score > 21)

Traumatic stress was predicted by loneliness (OR [95% CI] 1.32 [1.15; 1.53]), whereas being a man was protective (OR [95% CI] 0.29 [0.06; 0.97]).

#### Insomnia (ISI score > 14)

The predictors of insomnia were feelings of boredom (OR [95% CI] 7.57 [2.65; 23.99]), lifetime history of anxious disorder (OR [95% CI] 3.67 [1.61; 9.07]), and older age (OR [95% CI] 1.05 [1.02; 1.08]). Practicing sport every day was protective (OR [95% CI] 0.28 [0.08; 0.88]).

#### Psychological distress

The most predictive factor of psychological distress was a change in psychotropic drug use (OR [95% CI] 7.13 [2.46; 22.9]), followed by lifetime history of anxious disorder (OR [95% CI] 2.16 [1.02; 4.67]), and low frequency of leaving home (OR [95% CI] 2.46 [1.02; 6.16]). Practicing a physical activity was protective (OR [95% CI] 0.39 [0.16; 0.93] for several times/week, and OR [95% CI] 0.14 [0.04; 0.41] for every day).

## Discussion

This is the first study investigating psychological distress and its predictors during the first French national lockdown in a homogeneous sample of patients with a previous diagnosis of major depressive episode by a psychiatrist. This is different from most of the previous studies on COVID-19-related lockdowns that included patients with different psychiatric disorders, and mostly self-reported^5–10^. In our study, PP (patients with history of depression within the last 2 years) were more at risk to develop psychological symptoms during the lockdown than HC (without history of psychiatric disorders) who had very low levels of psychological symptoms, particularly depressive symptomatology and suicidal ideation. Similarly, other European studies showed that lockdown measures have a more important psychological impact in people who reported psychiatric disorder^12,14,18^. Compared with a Chinese study on 66 patients with anxious and/or depressive disorder diagnosed by a clinician, our analysis highlighted higher frequency of depression (35.5% of French vs. 23.6% of Chinese patients) and suicidal ideation (26.6% of French vs. 15.8% of Chinese patients), lower frequency of traumatic stress (15% of French vs. 31% of Chinese patients), and similar rates of anxiety and insomnia^15^.

Our analysis suggests that the presence of psychological symptoms was not related to living conditions and changes in habits during the lockdown because these variables were comparable between HC and PP. The limited access to care or healthcare interruption could have contributed to the higher risk of acute mental symptoms during the COVID-19 pandemic. Half of participants in the PP group did not have any contact with a psychiatric service since the lockdown initiation, for not specified reasons. In China, approximately 22% of patients reported a pandemic-related interruption of psychiatric care^19^. Moreover, in our study, changes in psychotropic use was a common predictor of all psychological outcomes. However, this change mostly consisted in an increase of psychotropic intake, which may be a consequence rather than a cause of the acute mental symptoms. Health professionals must find new methods to look after patients, and pro-active non-intrusive links with the healthcare system should be offered to people with pre-existing poor mental health. It is essential to provide continued psychiatric intervention through telepsychiatry, and to strengthen the patients social support via community mental health services.

It has been hypothesized that the pandemic might increase substance use in an attempt to cope with negative feelings^20^. In our sample, PP reported more frequently use of tobacco and cannabis than HC, who consumed more often alcohol. Approximately one third of substance users reported increased consumption. Similarly, in Spain, Solé et al.^14^ found that psychiatric patients increased the use of tobacco, but not of alcohol and cannabis, compared with controls. Moreover, a regular web-based survey of a representative sample of the French general population carried out by *Santé* *Publique* *France* (“Public Heath France”) to monitor health behaviors and mental health during the pandemics^21^ reported that one third of subjects increased tobacco consumption and 10% of subjects increased alcohol consumption associated with an initial increase in depression and anxiety levels after the lockdown implementation. Our analysis also found that alcohol use was predictive of anxious symptoms.

In our sample, lifetime anxious disorder was the only psychiatric disorder that predicted psychological distress. Indeed, people with anxiety-related disorders have been the most affected by the COVID-19 pandemic, with greater fears about several consequences^18^. In our study, loneliness and boredom were two common and independent predictors of depression and anxiety. Loneliness may be particularly prevalent and devastating during a pandemic, due to social distancing measures. At the onset of COVID-19, there has been concern about the effect of increased isolation on loneliness and other mental health conditions. A recent randomized controlled trial showed that a layperson-delivered, empathy-oriented short telephone call program reduces loneliness, depression, and anxiety and improves the general mental health of participants within 4 weeks^22^. Sense of isolation, confinement, reduced social and physical contacts can frequently cause boredom^23^. Boredom, a state that relates to low arousal with dissatisfaction due to perceived monotony and repetition^24^, is considered as one of the most relevant stressors in individuals who experienced isolation during the pandemic^25^. Perceived stress may influence emotional distress through boredom proneness^26^. People who are quarantined should be advised to stave off boredom and provided with practical advice on coping and stress management techniques^3^, such as mindfulness training^27^ or engaging in creative behaviors^28^. People should also be encouraged to regularly practice a physical activity that reduces boredom and the feeling of time slowing down during a lockdown^29^. In agreement, in our study, daily physical activity was a protective factor against depression, anxiety and insomnia. Besides its effect on boredom, regular physical activity might also reduce anxiety and depression^30^. The World Health Organization recommends 150 min of moderate intensity or 75 min of vigorous intensity physical activity per week, or a combination of both, during self-quarantine^31^. Our result might have been biased because only less anxious and depressed subjects were able to practice regular physical activity.

Another interesting result of our study is that virtual contacts were the only protective factor against suicidal ideation. This strengthens the need to propose individual-level interventions to reduce loneliness, enhance social support, and increase opportunities for social interactions. Psychological counselling telephone helplines and online consultations played a significant role in maintaining the citizens’ good mental health in China^32^. Telepsychiatry emergency services or hotlines should be made available to patients with intense suicidal ideation. In France, a brief contact intervention, including telephone calls (VIGILANS program), has shown its efficiency for reducing suicide reattempt^33^. The impact of such intervention deserves to be studied. Telemedicine provides new opportunities to address the patients’ mental health needs by creating disease awareness and improving treatment adherence^34,35^.

The present results must be interpreted in the light of some limitations. First, our findings may not be generalizable to all patients with depression. Participation in this study was on a voluntary basis and sampling was carried out online when strict lockdown measures were in place, thus excluding patients who did not have internet access. Second, the cross-sectional approach does not allow demonstrating a causality between self-perceived psychological status and lockdown. Future longitudinal studies should assess the consequences of the COVID-19 pandemic in psychiatric patients. Third, the current clinical characteristics were self-reported, but the lifetime psychopathology was diagnosed by a clinician before the lockdown.

To conclude, improved access to telepsychiatry services, home delivery of psychotropic medications, online psychiatric first-aid resources, and infectious disease outbreak preparedness play a pivotal role in minimizing the severity of psychiatric symptoms experienced by psychiatric patients. Our results might contribute to the development of specific strategies for mental health care by identifying potential targets of assessment and care in psychiatric patients, beyond the usual risk factors, such as loneliness and boredom. Mental health preparedness and anticipation of future outbreaks will lead to an increased awareness of the needs of psychiatric patients and of the contingency plans to be put in place.

## Material and methods

### Design and participants

This observational study was carried out the Department of Psychiatric Emergency and Acute Care of the Academic hospital of Montpellier, France. The adult participants have been previously included in research projects or followed at our department between March 15, 2019 and March 15, 2020. This allowed us to constitute two groups: HC and PP (out- and in-patients with history of depressive episode). Exclusion criteria were: absence of a previous psychopathology assessment using a validated psychometric tool (Mini-International Neuropsychiatric Interview, MINI; Structured Clinical Interview for the DSM-IV Axis I disorders, SCID-1; or Diagnostic Interview for Genetic Studies, DIGS), and refusal to participate.

In total, 69 HC without any history of psychiatric disorder and 346 patients with history of depressive episode within the last 2 years according to the DSM-IV criteria (PP) accepted to participate in this on-line survey. Lifetime psychopathology was previously assessed by a trained psychiatrist or psychologist using the MINI or DIGS.

The study protocol was registered in the Clinical Trials Registry (ClinicalTrials.gov NCT04374643) and was approved by the Institutional Review Board of Montpellier Academic Hospital (IRB-MTP_2020_12_202000421 (30/03/2020) and IRB-MTP_2020_12_202000436 (08/04/2020). All experimental methods were carried out in accordance with the ethical guidelines determined by the National Ministry of Health, Labour and Welfare and the Declaration of Helsinki. All participants provided written informed consent before entering the study.

### Procedure

During the French lockdown (March 17–May 11, 2020) an e-mail was sent to participants with an anonymization number and an information note about the study aim. In another e-mail, they received a computerized form to complete in which they needed to add their anonymization number.

### Outcomes

The study focused on the prevalence of six outcomes in the last 15 days: depression, anxiety, anger, insomnia, traumatic stress, and suicidal ideation. Depression symptoms and the presence of suicidal ideation were assessed with the PHQ-9^36^. A PHQ-9 score ≥ 10 has been associated with major depressive disorder (88% sensitivity and 88% specificity)^36,37^. The presence of suicidal ideation was assessed with the corresponding item of the PHQ-9 > 0 which evaluates the frequency of passive thoughts of death or self-injury within the last two weeks^38^, anxiety symptoms were measured with the GAD-7^38^. A GAD-7 score ≥ 10 corresponds to moderate to severe generalized anxiety disorder (89% sensitivity and 82% specificity)^39^. French versions of PHQ-9 and GAD-7 were freely downloadable on the patient Health Questionnaire website (www.phqscreeners.com). Anger state was assessed using the STAXI-state^40,41^. Distress resulting from a traumatic life event was evaluated with the 22-item IES-R40 using a cut-off of 21^42^, and insomnia with the ISI^43,44^ using a cut-off of 14 for clinical insomnia^45^. Moreover, a variable was created to describe psychological distress based on the presence of severe self-reported symptoms, i.e. presence of suicidal thoughts (suicidal item of the PHQ-9 > 0) or high score for at least one scale (PHQ-9 score > 9; GAD-7 score > 9; IES-R score > 21; ISI score > 14; STAXI-state above the median).

Regarding the factors associated with mental health outcomes and potentially related to the lockdown, sociodemographic data (gender, marital status, professional status), living conditions during the lockdown (living alone or not, home characteristics), habits during the lockdown [frequency of virtual (i.e. telephone and video) and written contacts (i.e. text messages, mails), feelings of loneliness and boredom, frequency of physical activity and going out from home, and their corresponding changes compared to usual life], COVID-19-related data (infectious status, fear of infection for the subject and relatives), access to psychiatric care, substance consumption (tobacco, alcohol, analgesics and psychotropic drugs) were collected and analyzed.

### Statistical analyses

The characteristics (psychological outcomes, socio-demographic variables, conditions of living and habits during the lockdown, COVID data) in the two groups (HC and PP) were described using medians (minimum–maximum) and numbers (frequency, percentages) for quantitative and qualitative variables, respectively. The significance of between-group differences was evaluated using the Kruskal–Wallis test, Fisher’s exact test, or Chi square tests. Cliff’s delta effect size and Odds Ratio were computed with their 95% confidence intervals for respectively quantitative and qualitative variables. P values were corrected for multiple testing using false discovery rate correction (FDR)^46^.

To manage the high number of variables evaluated as potential risk factors for psychological outcomes and their possible collinearity, a two-step variable selection was carried out for each outcome: (1) logistic regression was used to evaluate the crude relationships between the outcome and the potential risk factors from which only variables with p value < 0.15 were kept for step 2; (2) the retained variables were included in a multivariate logistic model and selected using the stepwise Akaike Information Criterion method (involving both forward and backward approaches) to keep only informative variables. Socio-demographic variables and history of psychiatric disorder were systematically kept in the model when analyzing the whole sample (HC and PP subjects). The same procedure was used also for PP alone. Variables describing habit changes were excluded from these analyses because they partly determined habits during the lockdown. Odds ratio (OR) and 95% confidence intervals (CI) were estimated for the best selected models. Significance of the associations might be estimated from the 95% CI. All analyses were performed with the R 4.0.3 software (R Core Team 2018, https://www.R-project.org/).
